# A RUL Estimation System from Clustered Run-to-Failure Degradation Signals

**DOI:** 10.3390/s22145323

**Published:** 2022-07-16

**Authors:** Anthony D. Cho, Rodrigo A. Carrasco, Gonzalo A. Ruz

**Affiliations:** 1 Faculty of Engineering and Sciences, Universidad Adolfo Ibáñez, Santiago 7941169, Chile; acholo@alumnos.uai.cl (A.D.C.); rax@uai.cl (R.A.C.); 2 Faculty of Sciences, Engineering and Technology, Universidad Mayor, Santiago 7500994, Chile; 3 School of Engineering, Pontificia Universidad Católica de Chile, Santiago 7820436, Chile; 4 Data Observatory Foundation, Santiago 7941169, Chile; 5 Center of Applied Ecology and Sustainability (CAPES), Santiago 8331150, Chile

**Keywords:** prognostics, fault detection, recurrent neural networks, prophet

## Abstract

The prognostics and health management disciplines provide an efficient solution to improve a system’s durability, taking advantage of its lifespan in functionality before a failure appears. Prognostics are performed to estimate the system or subsystem’s remaining useful life (RUL). This estimation can be used as a supply in decision-making within maintenance plans and procedures. This work focuses on prognostics by developing a recurrent neural network and a forecasting method called Prophet to measure the performance quality in RUL estimation. We apply this approach to degradation signals, which do not need to be monotonical. Finally, we test our system using data from new generation telescopes in real-world applications.

## 1. Introduction

Modern industry has evolved significantly in the past decades, building more complex systems with greater functionality. This evolution has added many sensors for better control, higher system reliability, and information availability. Given this improvement in data availability, new adequate maintenance policies can be developed [1]. Thus, maintenance policies have evolved from waiting to fix the system when a failure appears (known as reactive maintenance) to predictive maintenance, where intervention is scheduled with the information obtained from fault detection methods.

Various researchers confirm that sensors play a crucial role in preserving the proper functioning of the system or subsystem [2,3] as they provide information about the operating status in real-time such as possible failure patterns, level of degradation, abnormal states of operation, and others. Taking this into account, various methodologies have been developed for fault detection [4], testability design for fault diagnosis [5,6], detection of fault conditions malfunction using deep learning techniques [7,8], and test selection design for fault detection and isolation [9], just to name a few. Most of them share the same goal of being able to help increase the reliability, availability, and performance of a system.

The two main extensions of predictive maintenance are Condition Based Maintenance (CBM) and Prognostics and Health Management (PHM); both terms have been used as a substitute for predictive maintenance in the literature [10,11]. According to Jimenez et al. [11], they aligned these terms by adopting predictive maintenance as the first term to refer to a maintenance strategy, CBM as an extension of predictive maintenance, and adding alarms to warn when there is a fault in the system. Later, Vachtsevanos and Wang [12] introduced prognostics algorithms as tools for predicting the time-to-failure of components; from this insight emerged PHM [13] as an extension of CBM to improve the predictability and remaining useful life (RUL) estimation of a component in question after a fault appears. This information can then be used as a supply for decision-making in maintenance scheduling [14].

It is necessary to highlight that fault detection and prognostics are not always exclusive. Fault detection is usually an initial step in computing prognostics to estimate the future behavior of the system or subsystem.

Generally, faults are generated by degradation of the components that make up the system. Such degradation can be monitored through the signals collected from the sensors. There are various types of degradations that have been addressed in the literature, one of the most common are those signals that present degradation with slow decay that are present in different components, such as, for example, an increase in resistivity of fuses, reduction in currents on frequency processors, and the mean resolution of a telescope’s camera, among others. Considering these similarities, it is possible that an automatic fault detection framework that manages to detect the degradation in a frequency processor could also effectively detect the degradation in the resolution of a camera or vice versa. Similarly, it is possible that a good prediction of the RUL of a camera can be obtained using historical fault information present in other components.

This work focuses on prognostics by developing recurrent neural networks (RNNs) and a forecasting method called Prophet to measure the performance quality in RUL estimation. First, we apply this approach to degradation signals, which do not need to be monotonical, using the fault detection framework proposed in [15] with some improvements in the pre-processing and the cleaning data step. Later, we applied our approach to similar degradation problems but with different statistical characteristics.

The difference between our research with the rest of the works is in the scalability of the framework in fault detection towards other similar problems, showing its effectiveness and robustness. On the other hand, the adjusted RNN models with historical data of one type of fault to predict its RUL can also be used in other problems that have signals with similar degradation, such as the resolution of a telescope’s camera, showing the power of generalization and precision in the prediction of the RUL.

Our work has the following contributions:

1. We made improvements in cleaning spikes or possible outlines and smoothing time-series in the pre-processing data step in the fault detection framework developed in [15] to reduce the remaining noise level while maintaining its relevant characteristics such as trends and stationarity.
2. We show that the fault detection framework in [15], together with our pre-processing method, improves the robustness of the framework and can be transferable to another problem with similar degradation, although with different statistical characteristics.
3. We built a strategy using clustering run-to-failure critical segments to define an appropriate failure threshold that improves the RUL estimation. Moreover, using this strategy, we predict the RUL of another problem with similar degradation.

The rest of this article is organized as follows. First, the background related to this work is presented in Section 2. In Section 3, we present the proposed method for data pre-processing for cleaning spikes or outlier points, the smoothing for time series, and the process of prognostic for RUL estimation. In Section 4, the details of the application are given, as well as the results. Section 5 presents a discussion of results and performances obtained for each application. Finally, the conclusion of the work is presented in Section 6 and future work in Section 7.

## 2. Background

The following subsections present a brief description of fault detection, prognostics, performance measurements, and a method used for RUL estimation.

### 2.1. Fault Detection

Most modern industries are equipped with several sensors collecting process-related data to monitor the status of the process and discover faults arising in the system. Fault detection systems were developed around the 1970s [4,16], as an essential part of automatic control systems to maintain desirable performance. Fault detection can be defined as a process of determining if a system or subsystem has entered a mode different from the normal operating condition [15], and a fault may appear at an unknown time, and the speed of appearance may be different [17,18].

In the literature, a wide variety of methods used for fault detection can be classified into signal processing approaches [18,19,20,21,22,23], model-based approaches [23,24,25,26], knowledge-based approaches [18,27,28,29], and data-driven approaches [18,23,30,31,32,33,34,35,36]. With the arrival of technology and the advancement of computing methods, data-driven approaches are gaining attention in the last decades, where it is expected that the data will drive the identification of normal and faulty modes of operation. See [4] for a general description of fault detection and diagnosis systems.

Some recent developments have addressed this issue with deep learning to increase accuracy in fault detection. For example, Yao Li [37] presented a branched Long-Short Term Memory (LSTM) model with an attention mechanism to discriminate multiple states of a system showing high performance in its prediction based on the F1-score metric. On the other hand, Liu et al. [38] showed a strategy for failure prediction using the LSTM model in a multi-stage regression model to predict the trend; this is then used to classify the level of degradation by similarity with established failure profiles, achieving improvement estimates with better precision.

Zhu et al. [39] addressed the problem of classifying multiple states of a system with a convolutional network structure (CNN), specifically LeNet, optimized with Particle Swarm Optimization (PSO). Their results showed that this strategy achieves better performance and greater robustness compared to LeNet without PSO, VGG-11, VGG-13, VGG-16, AlexNet, and GoogleNet. Another approach using CNN is presented in the work of Jana et al. [40] which uses a suite of Convolutional Autoencoder (CAE) networks to detect each type of failure. Its design allows addressing failures in multiple sensors with multiple failures, obtaining an accuracy of around 99%.

Within the approaches not fully supervised, Long et al. [41] developed a Self-Adaptation Graph Attention Network, one of the first models of this type of network to be able to use a few-shot learning approach in which abundant data is available but very little is labeled and also to be able to incorporate cases of failures that rarely occur. In their results, they showed better performance at the level of accuracy compared to other models.

From an application perspective, fault detection systems have been developed in many areas such as rolling bearing, machines, industrial systems, mechatronics systems, industrial cyber-physical systems, and industrial-scale telescopes, to name a few [15,23,24,25,26,33,34,35,37,38,41,42].

Some of them describe some advantages and disadvantages over others in the applied methodology to obtain better results. However, there are still a lot of difficulties in implementing fault detection methods for real industries due to the properties of the data.

### 2.2. Prognostic

The prognosis task is mainly focused on estimating or predicting the RUL of a degrading system and reducing the system’s downtime [43]. So, the development of effective prognosis methods to anticipate the time of failure by estimating the RUL of a degrading system or subsystem would be useful for decision-making in maintenance [44]. A failure refers to the event or inoperable behavior in which the system or subsystem does not perform correctly.

According to the literature, prognostics approaches can be classified into model-based approaches [18,45], hybrid approaches [18,46,47], and data-driven approaches [18,48,49,50]. Data-driven approaches offer some advantages over the other approaches, especially when obtaining large and reliable historical data is easier than constructing physical models that require a deeper understanding of the system degradation. Also, they are increasingly applied to industrial system prognostic [18,44]. Recently, these studies are also divided into three branches: degradation state-based, regression-based, and pattern matching-based prognostics methods [51,52]. The former usually estimates the RUL by estimating the system’s health state and then using a failure threshold to compute the RUL. The second method is dedicated to predicting the evolution behavior of a degradation signal, and the estimation of the RUL can be obtained when the prediction reaches the failure threshold. The last methods consist of characterizing the signal and comparing it in the run-to-failure repository to compute the RUL by similarity.

In recent years, various deep learning models have been introduced to address forecasting problems in RUL prediction. For example, Kang et al. [53] developed a multilayer perceptron neural network (MLP) model to predict the health index of a signal; this is used in a polynomial interpolation model to estimate the RUL. They indicate that their strategy outperforms direct prediction methods using SVR, Linear Regression, and Random Forest. In an ensemble-type approach, Chen et al. [52] presented a hybrid method for RUL prediction using Support Vector Regression (SVR) and LSTM in which the results are functionally weighted, showing to be more robust as it takes advantage of the benefits provided by SVR and LSTM.

Among the most innovative methods, Ding and Jia [54] designed a convolutional Transformer network model that takes advantage of the attention mechanism and CNN to capture global information and local dependence of a signal allowing to directly map the raw signal to an estimated RUL, increasing its effectiveness and accuracy in prediction. On the other hand, Zhang et al. [55] developed a model that allows evaluating health status and predicting RUL simultaneously using a dual-task network model based on the bidirectional gated recurrent unit (BiGRU) and multigate mixture-of-experts (MMoE), resulting in better performance compared to traditional popular models such as ANN, RNN, LSTM, CNN, GRU and Bi-GRU, and with satisfactory higher robustness.

Under the not fully supervised approach, He et al. [56] developed a semi-supervised model based on a generative adversarial network (GAN) in regression mode, considering historical data for prevention and scarce historical information for failures to predict the RUL. This approach allows for avoiding overfitting, thus increasing its power of generalization and manages to achieve satisfactory accuracy even when the amount of historical data per failure is limited.

To measure the performance of the prognosis method, Saxena et al. [57] introduced some standard evaluation metrics that were used to evaluate several algorithms compared to other conventional metrics effectively. Such metrics can be used as a guideline for choosing one model over another. A description of these metrics can be found in Appendix A; they can be considered as a hierarchical validation approach for model selection described in [57], where the first instance is to check out whether a model gives a sufficient prognostic horizon, and if not, this method is not meant to compute the other metrics. If the model passes PH’s criterion, it is followed by the computation of the α–λ accuracy, which needs a more strict requirement of staying within a converging cone of error margin as a system reaches the End-of-Life (EoL). If this criterion is also met, we can quantify how well the method does by computing the accuracy levels relative to the actual RUL and, finally, measure how fast the method converges. This work will focus on the first two metrics since they provide a meaningful level of accuracy of the model in the RUL estimation.

### 2.3. Recurrent Neural Networks (RNNs)

Among data-driven techniques used for prognostics, RNNs have been widely studied in recent years and are one of the most powerful tools as they can model significant nonlinear dynamical time series. A large dynamic memory is allowed to preserve temporal dynamics of complex sequential information and has been used with success in several prognostic applications [49]. Three types of RNN are chosen in this work: Echo State Networks (ESNs), Long-Short Term Memory (LSTM), and Gated Recurrent Unit (GRU), to measure the performance of RUL estimation applied in three problems described in Section 4. A description of these RNNs appears in Appendix B.

### 2.4. Prophet Model

The Prophet model was developed by Sean Taylor and Benjamin Letham [58] in 2018 to produce more confident forecasts. Its methodology consists of the usage of a decomposable time series model, consisting of three main components: trend, seasonality, and holidays. It allows one to look at each component of the forecast separately. These components are combined as an additive model in the following form:

(1) y(t)=g(t)+s(t)+h(t)+ϵ(t),

where g(t) is the trend function that represents the non-periodic changes of the time series, s(t) describes the periodic changes (daily, weekly, and yearly seasonality), h(t) defines the effects of holidays that occur on potentially irregular calendar schedules over one or more days, and ϵ(t) represents the error term of any idiosyncratic changes which are not accommodated by the model. This method has several advantages that allow the analyst to make different assumptions about the trend, seasonality, and holidays if necessary, and the parameters of the model are easy to interpret.

## 3. Methodology

### 3.1. Pre-Processing Data

**Figure 1:**
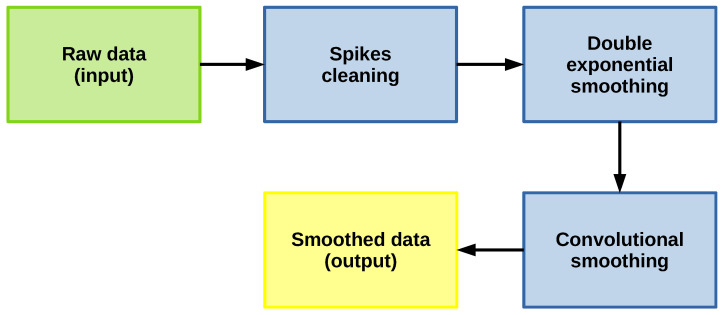
Pre-processing flow chart.

The data or signals collected from a system, in most cases, are noisy, and some outliers or spikes might be present. So, it is necessary to pre-process each signal before feeding it to the forecasting model. This process is shown in Figure 1, and it consists of the following steps:

1. **Spikes cleaning**: it consists of clearing possible outliers and spikes points by comparing time series values with the values of their preceding time window, identifying a time point as anomalous if the change of value from its preceding average or median is anomalously large.
  An advantage of this outlier reduction strategy is that it considers the local dynamics of the signal with time windows. Therefore, managing to identify as outliers the samples that are outside the local range and thus reduce the number of samples that are normal but that were identified as outliers, as could happen with traditional methods that depend on the global mean and standard deviation. This method is implemented in the ADTK library [59].
2. **Double exponential smoothing**: this filter [26,60,61,62,63,64] is commonly used for forecasting in time series, but it can also be used for noise reduction. This method is particularly useful in time series to smooth its behavior, preserving the trend and without losing almost any information in the dynamics of the series. Also, the model is simple to implement, depending on two main parameters. For more details, see [15].
3. **Convolutional smoothing**: this consist of applying the Fourier transform with a fixed window size to smooth the signal maintaining the trend. In other words, this method applies a central weighted moving average to the signal allowing short-term fluctuations to be reduced and long-term trends to be highlighted. It is implemented in the TSmoothie library [65].

**Figure 2:**
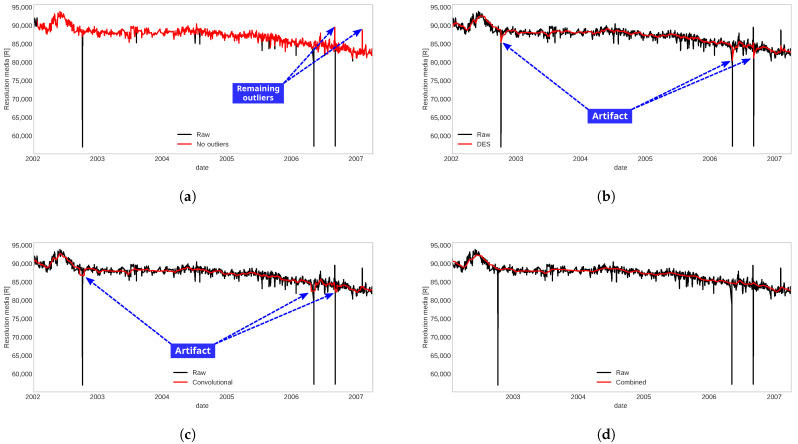
Application of each method separately to the raw signal. (**a**) Outliers and spikes cleaning. (**b**) Double Exponential Smoothing. (**c**) Convolutional smoothing. (**d**) Proposed pre-processing method.

Each of the methods that make up the pre-processing process offers some strengths and weaknesses. To see its independent effect, each of the methods was applied to a signal that presented outliers with a high level of noise, as shown in Figure 2.

The effect of the method that was mentioned in Step 1, shown in Figure 2a, can be seen that it manages to reduce the large jumps that are considered outliers, but still, some outliers remain with minor jumps. The noise reduction or smoothing methods that were mentioned in Steps 2 and 3 present some artifacts in the signal dynamics due to outliers, and their effects are unknown, as shown in Figure 2b,c.

It is for this reason that we combine the methods to use the advantages offered by each one of them, allowing us to reduce large jump outliers, followed by a noise reduction strategy and reduce minor jump outliers, and finally, reduce possible remaining artifacts with smoothing procedure as presented in the designed pre-processing scheme, Figure 1. The effect of this combination is shown in Figure 2d, where the resulting signal has smoother dynamics and preserves the trend of the original signal.

### 3.2. Run-to-Failures Critical Segments Clustering

The increase in processor speed, sensors monitoring, and the development of storage technologies allow real-world applications to record changing data over time easily in components of a system/subsystem [66]. It is necessary to highlight that the components used in different environments lead to different degradation levels, even for one type of component. Therefore, the failure threshold can be different in each situation. However, from the historical run-to-failure signals, they can be clustered so that each signal in a cluster behaves similarly; thus, it is possible to define a failure threshold based on the signals that belong to a cluster. In other words, there is a failure threshold A that can be defined as cluster A, a failure threshold B to cluster B, and so on.

Our scheme of clustering does not consider the entire signal since it starts running until EoL; instead, we use the critical segment of the signal for clustering. Our definition of a critical segment of a signal is the segment where the degradation begins until EoL. Under these critical segments, we build clusters so that each cluster has signals with a degradation level relatively similar.

The advantage of clustering by critical segments allows us to define, in an easy way, the different failure thresholds. Therefore, we can define for each cluster an appropriate failure threshold based on the critical segment signals that belong to a cluster. To increase the effectivity, each critical segment is centered with its own standard normal condition value before the clustering process, i.e, if *S* is the complete signal, and $S^{\prime }$ is the critical segment, then $S^{\prime }$ is centered by $S^{\prime }-S_{0}+k$, where *k* is the standard normal condition value and $S_{0}$ is the first sample of *S*. Lastly, a threshold can be defined as the minimum degradation level reached by critical signals in the cluster.

### 3.3. Prognostic Method

Two strategies are proposed to deal with the estimation of RUL in components. For all strategies, we consider the fault date as the point in time $t_{P}$ at which the fault prediction starts [67]. We also assume that the recollected data consists of daily samples, which were processed using the approach presented in Section 3.1. In what follows, a description of these strategies is presented.

#### 3.3.1. Strategy A

**Figure 3:**
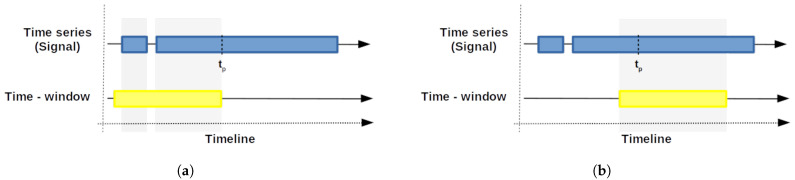
Time-window examples. (**a**) Time-window with missing values. (**b**) Time-window without missing values.

This strategy is based on a regression model, similar to the prognostic approach proposed in [48]. In this strategy, we define a time window of *d* days in which we analyze the data. Note that the number of samples in the time window can vary since data is not assumed to be available every day. Figure 3a shows an example with missing data, whereas Figure 3b shows an example where data is available through the whole time window.

**Figure 4:**
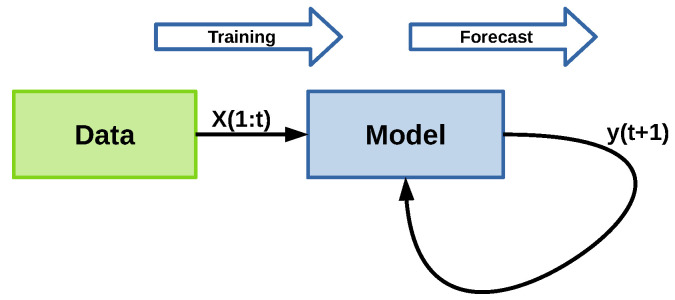
Model training and forecast structure.

The data within the time window is used to train the model, which is then utilized to predict a forecast for the next *n* days, following the structure shown in Figure 4. In this approach, X(1:t) represents the first *t* samples of *X*, the data used as input to train the model. The model then estimates y(t+1), and the current window is updated by dropping the oldest value and adding the newly calculated one: [X(2:t),y(t+1)]. The forecasting process is similar to the P-method developed in [48].

Using the previous forecast, we verify if the failure threshold is crossed within the time window, calculating the RUL if this occurs. This procedure is applied in a rolling window fashion whenever new data arrives.

**Figure 5:**
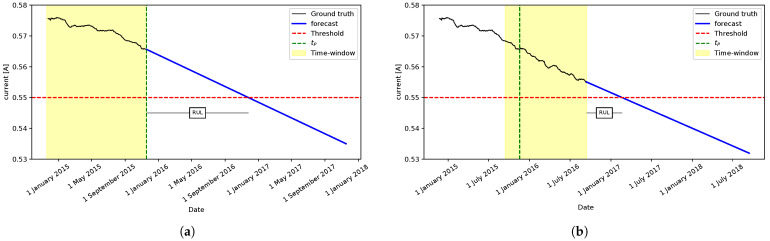
An example of RUL estimation using a time-window size of 365 days. (**a**) Time-window samples until fault date $t_{P}$. (**b**) Time-window shifted by 300 days.

**Figure 6:**
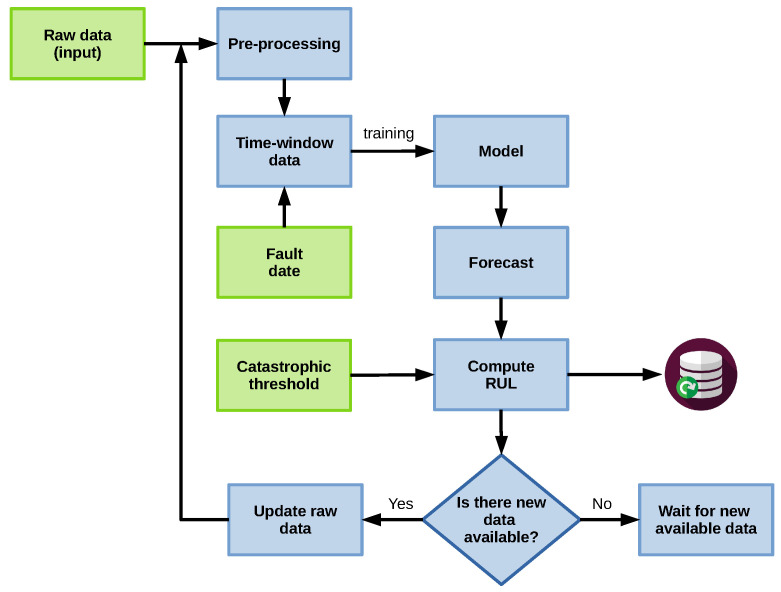
Prognostic process: strategy A.

Figure 5 shows an application example using a time window of 365 days. The first iteration result is shown in Figure 5a, with the time window between 18 November 2014 and 18 November 2015. Since some data is missing, we have 340 samples in this case. In this step, our approach estimates the RUL to be 384 days. Next, Figure 5b shows the results of the second iteration, where the time window lies between 14 September 2015 and 13 September 2016, containing 365 samples. In this step, the RUL is estimated to be 181 days. The black line represents the ground truth in both figures, and the blue line represents the obtained forecast. The green dashed line is $t_{P}$, the red dashed one is the failure threshold, and the RUL value is computed as the difference between when the forecast crosses the failure threshold and $t_{P}$. Finally, the whole process is shown in the diagram in Figure 6.

#### 3.3.2. Strategy B

**Figure 7:**
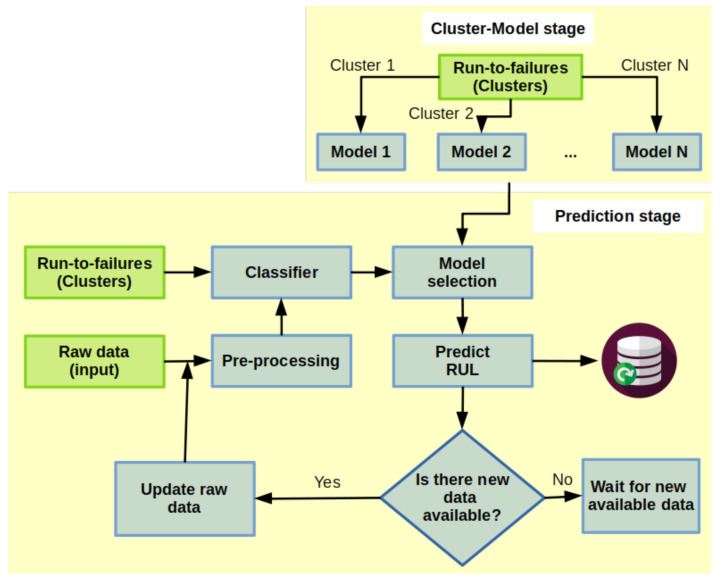
Prognostic process: strategy B.

Considering that one type of component could be in vastly different environments, it is possible that their degradation level, and thus failure thresholds, could be very different. Due to this, we need to adapt the previous strategy to account for this difference. We do this by combining matching and regression-based methods. This technique consists of two steps:

- **Cluster-Model stage**: it consists of the usage of clustering described in Section 3.2, so that, for each cluster we can fit a regression model. The train data is defined by the critical signals limited by a defined failure threshold in the cluster with its residual RUL, i.e., for each critical signal *S* with length l(S) in cluster *C* and $S^{\prime }\subset S$ such that $S_{0}^{\prime }=S_{0}$, and $S_{l(S^{\prime })}^{\prime }\approx failure\_threshold$. Then, each sample $S_{i}^{\prime }\in S^{\prime }$ has a residual RUL
  $$r_{i}:=Normalize\left(S_{i}^{\prime }\right)\cdot l\left(S^{\prime }\right),$$
  where l(S) is the length of the signal *S*,
  $$Normalize\left(S_{i}\right)=\frac{S_{i}-min\left(S\right)}{max(S)-min(S)},$$
  $S_{0}$ and $S_{0}^{\prime }$ are the first sample of *S* and $S^{\prime }$, respectively.
- **Prediction stage**: it consists mainly in predicting the RUL of a component in the signal that has been diagnosed as a fault, which means a degradation behavior has started. In this step, we took a segment of the signal after a fault has been detected; it is pre-processed and submitted to a classifier to identify to which cluster it belongs and select the related regression model, already fitted in the Cluster-Model stage, to predict the RUL. This procedure is executed when new samples are available.
  The classifier works in matching segments to all run-to-failure critical segments using Minimum Variance Matching (MVM) [68,69,70], which is a popular method for elastic matching of two sequences of different lengths by mapping the problem of the best matching subsequence to the problem of the shortest path in a directed acyclic graph providing the minimum distance. The classification scope provides the assignment by a voting criterion, i.e., the maximum number of signals of a cluster closer to a given segment will be taken. A flow chart of this prognostic process is shown in Figure 7.

The principal models used in this work for training and computing forecasts or RUL are mentioned in Section 2.3 and Section 2.4: ESN, LTSM, GRU, and Prophet (only for Prognostic Strategy A). To measure how well the model is for estimating RUL, we will use the prognostic horizon and α–λ accuracy.

## 4. Application Setting

### 4.1. Crack Growth

The crack propagation description is one of the most important components in the analysis of the life span of structural components, but it may require time and expense to investigate experimentally [71]. Hence, the estimation of crack propagation and durability of construction or structural component will be useful to estimate the remaining life of the component.

#### 4.1.1. Problem Description

As described in [72,73,74], components that are subjected to fluctuating loads are practically found everywhere: vehicles and other machinery that contain rotating axles and gears, pressure vessels and piping may be subjected to pressure fluctuations or repeated temperature changes, and structural members in bridges are subjected to traffic loads and wind loads, and some other applications. If the components are subjected to a fluctuating load of a certain magnitude for a sufficient amount of time, it may cause small cracks in the material. Over time, the cracks will propagate up to the point where the remaining cross-section of the component cannot carry the load, at which the component will be subjected to sudden fracture. This process is called fatigue and is one of the main causes of failures in structural and mechanical components.

**Figure 8:**
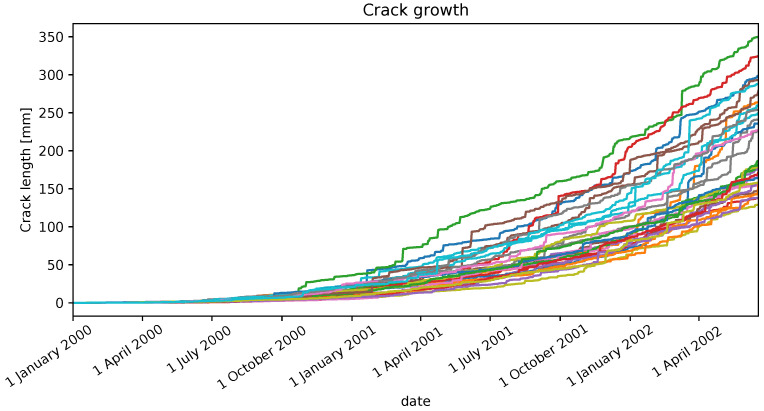
30 crack growth trajectories.

The common Paris–Erdogan model is adopted [72] for describing the evolution of the crack length *x* as a function of the load cycles *N* summarized by the following discrete-time model

(2) $$x_{t+1}=x_{t}+Ce^{\omega_{t}}{\left(\beta \sqrt{x_{t}}\right)}^{n},$$

where $\omega_{t}∼N\left(0,\sigma_{w}^{2}\right)$ is a random variable depicting white Gaussian noise, and *C*, β and *n* are fixed constants. A generation of 30 crack growth trajectories using Equation (2) is illustrated in Figure 8 and consists of 900 days of samples per trajectory.

#### 4.1.2. Prognostic

For practical purposes, we choose one trajectory from Figure 8 to estimate RUL to measure the performances of both strategies.

**Figure 9:**
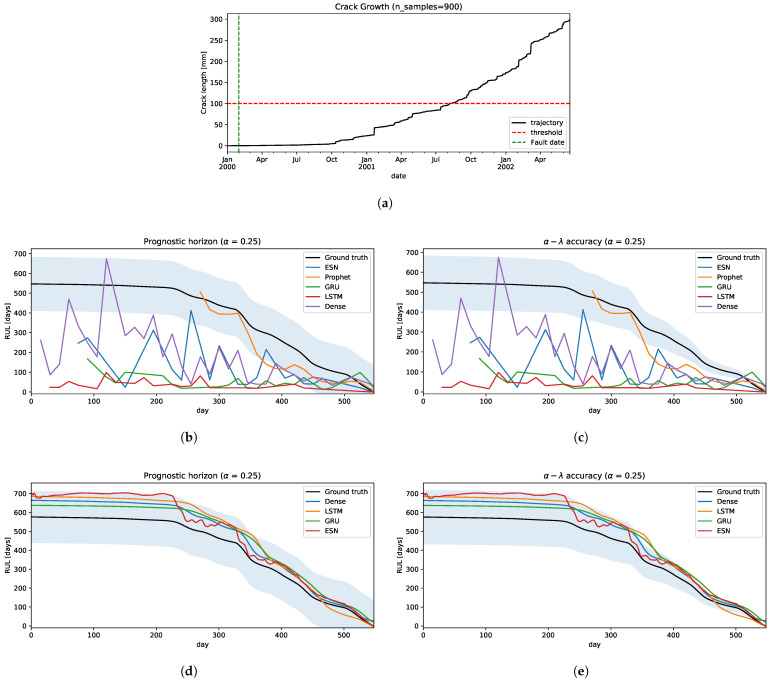
The crack growth prognostic. (**a**) Testing: a crack growth trajectory. (**b**) Strategy A: the prognostic horizon metric. (**c**) Strategy A: the α–λ accuracy metric. (**d**) Strategy B: the prognostic horizon metric. (**e**) Strategy B: the α–λ accuracy metric.

- Strategy A: following the methodology in Section 3.3.1, we estimate RUL shifting the time window by 15 days in every iteration, 1 year size of time-window, and 2 years of forecast.
  The results are shown in Figure 9. In the prognostic horizon, Figure 9b, we can see that all the models underestimate RUL, with some exceptions like the Dense neural network model. Neural network models had poor performances of RUL estimation and mostly fall outside of the confidence interval. Only the Prophet model is relatively close to the ground truth RUL. Concerning the α–λ accuracy, only Prophet has a segment close to the ground truth but then falls outside of the confidence interval, underestimating the RUL.
- Strategy B: using the technique proposed in Section 3.3.2 in this problem, we will simplify some steps of this process. Given that all the degradation trajectories are similar, we can assume only one cluster and the classifier will assign to it every time. Hence, the Cluster-Model stage has only one model, which is used to predict the RUL. Basically, this scheme becomes a simple regression model where it is fitted with all the historical-critical segment trajectories limited by its failure threshold and its residual RUL. We use 100 trajectories as run-to-failure signals generated from Equation (2) to fit the model.
  The performances can be seen in Figure 9d,e. All the models fall inside the confidence interval in the prognostic horizon and are getting closer to the ground truth as they reach the EoL, as illustrated in Figure 9d. Similar behavior is obtained for α–λ accuracy, as shown in Figure 9d. Only a few times, some methods go out and then go back into the confidence interval, e.g., LSTM and GRU, but these behaviors are acceptable.

The results are shown to indicate a large difference in the estimation of the RUL between the two strategies. This is due to the fact that the models that use strategy A are more sensitive to small variations in the signal, making the EoL estimate highly variable and, most critically, it is unaware of the possible variation that it may present in the future. On the other hand, the models that use strategy B take advantage of historical information to incorporate into the model information on how the signal could evolve, reducing the sensitivity due to small disturbances and better mapping to a more precise RUL.

### 4.2. Intermediate Frequency Processor Degradation Problem

The Atacama Large Millimeter/submillimeter Array (ALMA) is a revolutionary instrument operating in northern Chile’s Atacama desert’s very thin and dry air at an altitude of 5200 m above sea level. ALMA is one of the first industrial-scale new generation telescopes, composed of an array of 66 high-precision antennas working together at the millimeter and submillimeter wavelengths, corresponding to frequencies from about 30 to 950 GHz. Adding to the observatory’s complexity, these 7 and 12-m parabolic antennas, with extremely precise surfaces, can be moved around at the high altitude of the Chajnantor plateau to provide different array configurations, ranging in size from about 150 m to up to 20 km. The ALMA Observatory is an international partnership between Europe, North America, and Japan, in cooperation with the Republic of Chile [75].

#### 4.2.1. Problem Description

The Intermediate Frequency Processor (IFP) of the antennas of the ALMA telescope, as described in [25], is a critical component responsible for the second down-conversion, signal filtering, and amplification of the total power measurement of sidebands and basebands. This subsystem allows for effective communication of the captured data to the central correlator for processing, thus making it a central and critical component of each antenna. It is necessary to highlight that there are 2 IFPs per antenna, one for each polarization, and each IFP has sensors measuring currents of three different voltage levels: 6.5, 8, and 10 volts. For 6.5 and 8 volts, currents have four different basebands: A, B, C, and D, whereas, for 10 volts, sidebands USB and LSB, and switch matrices SW1 and SW2 currents are read. Each current is sampled every 10 min.

One of the diagnosed degradation problems that occur in the IFP module is due to hydrogen poisoning caused by hydrogen outgassing in tightly sealed packages [25], where this degradation can be tracked by monitoring current signals collected from each module.

#### 4.2.2. Prognostic

**Figure 10:**
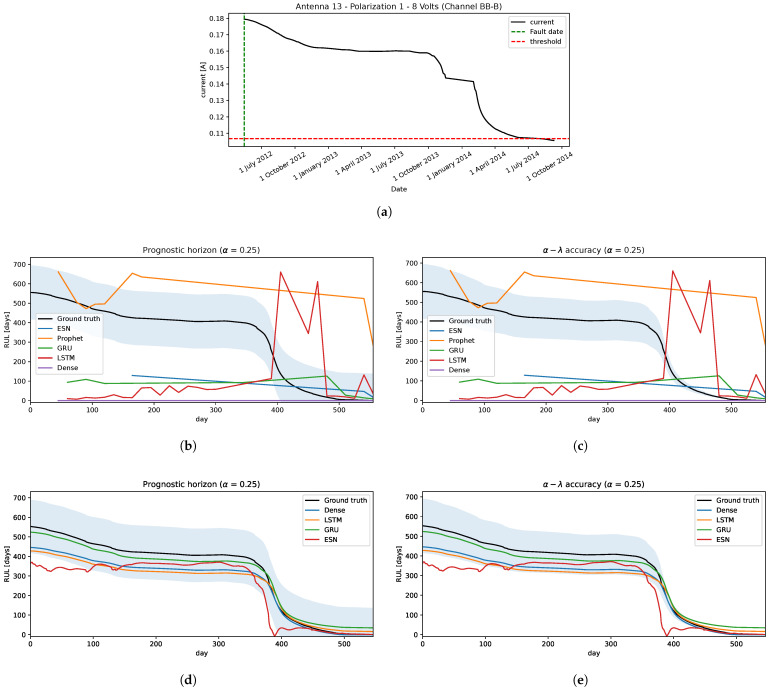
The IFP prognostic. (**a**) Testing: a signal from an IFP. (**b**) Strategy A: the prognostic horizon metric. (**c**) Strategy A: the α–λ accuracy metric. (**d**) Strategy B: the prognostic horizon metric. (**e**) Strategy B: the α–λ accuracy metric.

To measure the performance of both strategies, we selected one of the signals with a fault detected in [15], and applied the data pre-processing. This is shown in Figure 10a.

**Figure 11:**
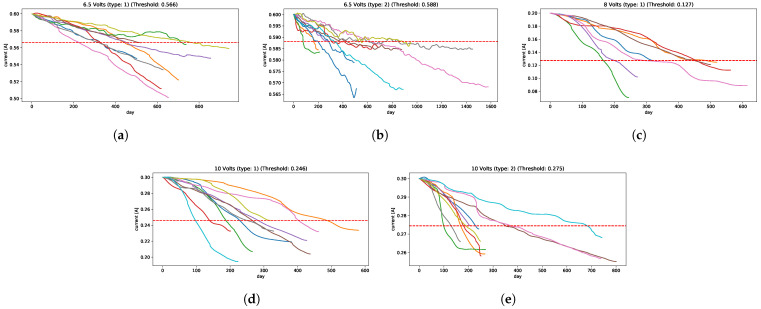
The IFP signals clustering, the red dashed lines represent the failure threshold defined for each cluster, and continuous lines are the critical segments segmented from the run-to-failure IFP signals (**a**) Class 1: 6.5 Volts (Degradation type 1). (**b**) Class 2: 6.5 Volts (Degradation type 2). (**c**) Class 3: 8 Volts. (**d**) Class 4: 10 Volts (Degradation type 1). (**e**) Class 5: 10 Volts (Degradation type 2).

- Strategy A: the performances of this method are illustrated in Figure 10b,c, in which we can see that none of these models give good predictions of RUL, nor when it approaches the EoL.
- Strategy B: from the historical run-to-failure signals, different degradation levels appears in each voltage’s current of the IFP. In this application, each voltage’s signals are clustered into a few clusters so that signals in each cluster have similar degradation levels making it easier to define an appropriate failure threshold in each cluster, just as described in Section 3.2, defining a total of 5 clusters for this problem: 2 cluster for 6.5 volts, 1 cluster for 8 volts, and 2 clusters for 10 volts; they are shown in Figure 11, in which, for each cluster has its corresponded failure threshold value, i.e., 0.566 is the failure threshold for cluster 1, 0.2 for cluster 2, 0.127 for cluster 3, 0.246 for cluster 4, and 0.275 for cluster 5; or it can be explained as 5.7%, 2%, 36%, 18%, and 8.3% of degradation levels for each cluster, respectively. These clusters are used to classify the new arriving pre-processed signal to select the appropriate failure threshold and predict the RUL.
  The cluster generation criterion focuses mainly on the Minimum Variance Matching (MVM) similarity metric, which is obtained by solving a shortest path (SP) problem that measures the distance between pairs of signals. The principle is to fix a signal as a centroid and compute the distances with the other signals; these distances are ordered, and using the same fundamentals of the elbow method, a group of signals is selected to form a cluster $C_{1}$ and the rest in another group $C_{2}$. This process is repeated for the cluster $C_{2}$ to verify if the signals are similar or if another cluster is generated, and so on. Repeated runs were made, resulting in most cases with 5 clusters being enough to separate these signals.
  The performances under both metrics, Figure 10d,e, show that almost all models have relatively good predictions of RUL falling inside of the confidence interval. Only ESN has some irregularities, but these underestimations are acceptable. The Dense neural network model outperforms the others slightly when it gets close to the EoL.
  Analyzing the results, the models that used strategy A showed a problem similar to what occurs in the application of the Crack Growth in Section 4.1.2, in which the models remain sensitive to small variations, generating a great variability in the estimation of EoL and therefore, affects the prediction of the RUL.
  Taking into account these effects that it could have on the models, if strategy B is used and a set of historical run-to-failure signals is considered that have great variability in the degradation behavior, different from that used in Section 4.1.2 in which the signals are quite similar, could affect the models in predicting the RUL due to these variations in the level of degradation of the historical signals.
  To avoid this, it was decided to group the signals into groups that are similar in degradation level and address them separately. As a consequence, the performance in different models manages to predict the RUL close to the real value.

### 4.3. Validation in a Different Setting

To validate our approach, we considered testing this methodology in a very different setting. In particular, we used measurements of camera resolution information from an important optical telescope.

#### 4.3.1. Problem Description

One of the problems presented in the studied instrument is the Teflon wear in the lens support, increasing the humidity level, which affects the camera resolution. This degradation can be tracked through measurements collected from the camera’s CCDs.

**Figure 12:**
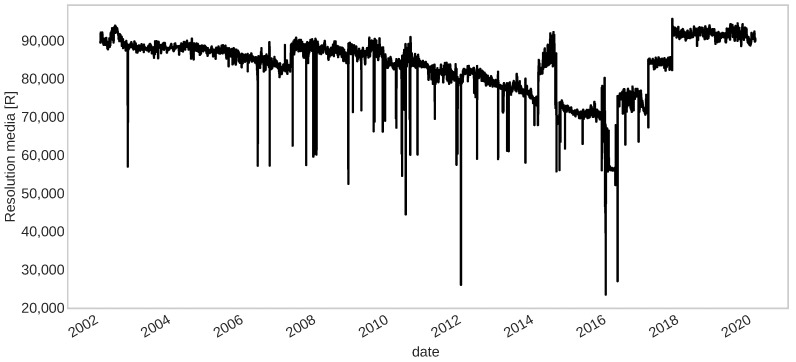
Resolution media signal obtained from a CCD.

An example of degradation over 18 years is shown in Figure 12, where it can be seen that this signal is noisy and has several spike points (large down jumps that may be possible outliers). Some corrective or maintenance actions have been made (time indexes of up jumps) are taken along these records. Therefore, a process of fault detection would be excellent for anticipating an unacceptable deviation of the fault-free behavior and then a prognostic process to compute the RUL of the component accurately.

#### 4.3.2. Fault Detection

**Figure 13:**
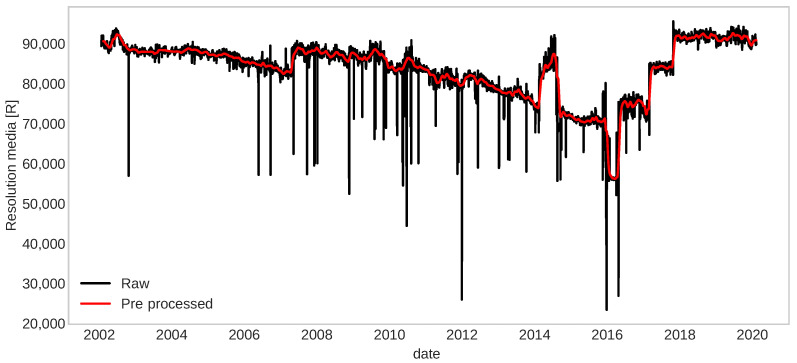
Raw and pre-processed signal of the resolution media obtained from a CCD.

Recently, Cho et al. [15] tackled similar degradation noisy signals using a fault detection framework based on ESNs applied to IFPs of the antennas of the ALMA observatory; the authors highlighted the noise level in the data affected the performance of detection significantly. In the case of the camera resolution, unlike the ALMA IFP data, it contains larger spikes that distort the signal dynamics even after double exponential smoothing. For this reason, it is necessary to adopt a mechanism that allows reducing spikes efficiently in time series as a clean outlier method in the pre-processing stage of the framework proposed in [15]. With this insight, the modified data pre-processing method was generated, and it is described in Section 3.1. The results, applying the proposed data pre-processing method, are shown in Figure 13, where the red signal represents the pre-processed signal, and the trend is maintained from the raw signal.

**Figure 14:**
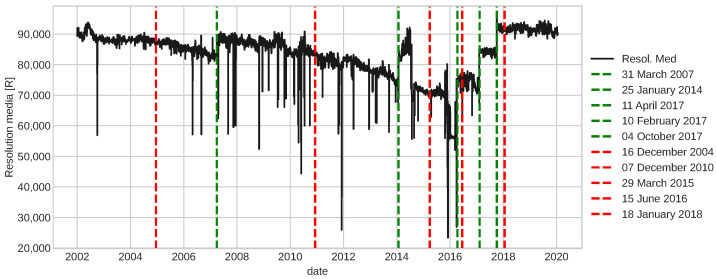
Fault detection in the resolution media signal obtained from a CCD.

Once the pre-processing stage is done, the fault detection process is maintained almost the same as in [15]. The result is shown in Figure 14. The vertical dashed red lines are fault detected time indexes and the vertical dashed green lines are time indexes where corrective or maintenance were made.

It is necessary to highlight that the framework designed in [15] deals with current signals with a resolution of 10 min per sample, resulting in high performance on real data. Now, with this modification in the pre-processing, the robustness of the framework increases, and it is applied to the camera problem, which are signals coming from a resolution camera with daily samples, resulting in the same effectiveness in fault detection; this is justified in that the degradation characteristic is similar to the ones that were used during the design of the method.

#### 4.3.3. Prognostic

**Figure 15:**
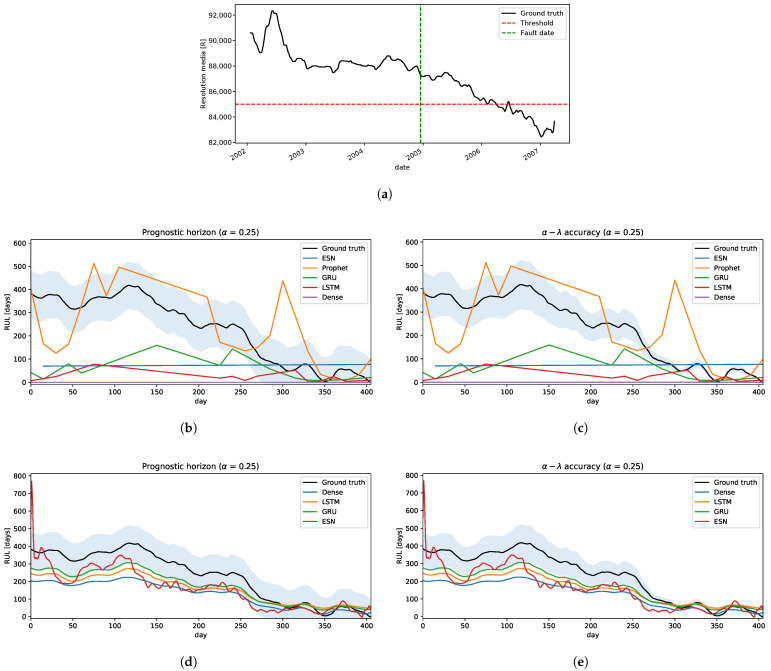
The Camera Resolution prognostic. (**a**) Testing: Resolution media trajectory. (**b**) Strategy A: The prognostic horizon metric. (**c**) Strategy A: The α–λ accuracy metric. (**d**) Strategy B: The prognostic horizon metric. (**e**) Strategy B: The α–λ accuracy metric.

For the prognostic application to the camera resolution signal, we took the first segment of the trajectory until the first maintenance, dated 2007-03-31, as the test signal for RUL estimation, Figure 15a. The rest of the segment can be computed similarly by applying the methodology described in Section 3.

- Strategy A: applying this method, we can see Figure 15b,c, that neural networks have a poor quality of predictions, whereas the Prophet model has some segments that fall inside the confidence interval, but it is not good enough because of its irregular behaviour.
- Strategy B: in this problem, there are no historical run-to-failure signals. So, clustering over this component is not possible. However, given that the degradation behavior present in this component is similar to the IFP of ALMA, we can use these clusters and try to transfer to this problem. To achieve this, it is necessary to transform the new arriving pre-processed signal *Q* and scale it to every cluster described in Section 3.2, this means, for each cluster, we define a transformed signal of *Q* as follows
  (3) $$\begin{array}{ccc} S & = & \kappa \cdot Q, \end{array}$$
  (4) $$\begin{array}{ccc} S^{\prime } & = & S-S_{0}+k_{i} \end{array}$$
  where,
  (5) $$\kappa =\frac{k_{i}-k_{i}^{*}}{Q_{0}-q^{*}}$$
  is the scaling constant, $k_{i}$ and $k_{i}^{*}$ are standard normal conditions and failure threshold of the cluster *i*, respectively. $Q_{0}$ is the first sample of the signal in this problem, and $q^{*}$ is its associated failure threshold.
  The classifier result gives the final scope, which is used for model selection in the prediction of RUL. In the prognostic horizon metric, Figure 15d, the GRU model outperforms the other models. However, the other models fall inside the confidence interval after 200 days. So, all the models in this metric are acceptable. From the α–λ accuracy side, most of the time, these models are not inside the confidence interval, underestimating the RUL on the first 300 days (λ=3/4). After that, they are around the ground truth up to the EoL. In this case, the GRU model is close to the frontier of the confidence interval, which is not as bad as an instance for RUL computation by using a similar degradation signal developed from another system or component like the IFP Problem.

The way in which strategy B was approached in this application allows comparing the critical segment of the new pre-processed and transformed incoming signal with the clustered signals that have similar patterns at the level of degradation. In addition, this helps to relate to possible trajectories of the signals of the cluster that is most assimilated and, thus, to be able to approximate the RUL of this new signal when historical information is not available. As the mean resolution signal has similar characteristics to some signals in one of the clusters, this helps in obtaining a relatively good RUL prediction.

## 5. Discussion

Several frameworks of fault detection have been developed in the last decades, most of them for a specific degradation present in an application of interest. In this work, we are interested in a more general framework, transferable to many domains that present a similar degradation problem. In Section 4.3.2, we show that the fault detection framework developed in [15] can be transferable to other applications with similar degradation behavior as the one described in Section 4.3.1, without any adjustment to the structure but only some improvement to the data pre-processing step. In particular, by adding other properties of noise to get a better-smoothed signal, as the example shown in Figure 13. Such improvement increases the performance of this framework slightly even when applied to the IFP signals, which was the problem of interest in [15]. We obtained a smoothed signal while maintaining the relevant characteristic of the raw data, such as the degradation trend. This smoothed signal then was used as an input to verify if a fault was present and returned the date where it was detected, as illustrated in Figure 14, where the red dashed lines represent the dates of detected faults and the green ones represent the dates of the performed maintenance.

The parameters used in the pre-processing steps were: Factor used to determine the bound of the normal range based on the historical interquartile range was fixed as 3, and the window size was fixed as 20 for both spike cleaner and convolutional smoothing methods.

It is necessary to highlight our meaning of transferable is not the same as transfer learning used in the context of deep learning. The framework learns from the data automatically but does not inherit the insights from another problem so that it can be scaled and applied to other similar problems. Given that fault detection and prognostic are not always exclusive to each other, in most of the cases, the former is considered as the previous step of the prognostic process. Additionally, the pre-processing method that we designed in Section 3.1 allows us to reduce as far as possible problems of outliers present in the signal to be later used, either for fault prediction or forecasting. This allows to increase the performance and reduce possible disturbances that affect the estimation.

For prognostic settings:

- Strategy A: time-window size was 365 days, 2 years of forecasting, a lookback of 19 samples format (e.g., samples from time t−19 until time *t* with a total of 20 samples) as input, and 20 epochs for neural networks adjustments. For simplicity, we assume for this method that new data is available every 15 days to update RUL estimation. The model hyperparameters used for prognostics are summarized in Table 1.
- Strategy B: a lookback of 9 samples format (e.g., samples from time t−9 until time *t* with a total of 10 samples) as input, and 15 epochs for neural networks adjustments. The model hyperparameters used for prognostics are summarized in Table 2.

All the algorithms were implemented in Python version 3.8.5 and ran on a computer with an Intel® Core™ Processor i5-3230M of 2.6 GHz × 4 cores, with 8 GB RAM, and using Linux Mint 20.1 Ulyssa (64 bits) as OS.

Two prognostic strategies were tested in three problems:

- Crack Growth in Section 4.1.2: is a classical problem in the literature in which the degradation is a monotonical non-decreasing trajectory. The worst performances are given by strategy A, where only the Prophet model was relatively close to the ground truth RUL. Whereas, the strategy B, all prediction models are significantly well performed on both metrics.
- IFP Degradation in Section 4.2.2: the historical degradation signals are not totally monotonous with different degradation levels and speeds, resulting in different failure threshold values for a set of signals. With this insight, defining a unique failure threshold for all the signals and forecasting the dynamic of the signal until reaching the failure threshold as described by strategy A does not work well. Therefore, clustering signals by degradation levels helps to define appropriately the failure threshold given the characteristic of similarity to a set of historical run-to-failure signals from a cluster. Therefore, using strategy B improves the prediction of RULs, in which ESN is the less accurate model than the other models tested.
- Camera Resolution Degradation in Section 4.3.3: the degradation trajectory showed irregularities similar to the IFP signals, in which there is some segment increase and then decrease, and vice versa. Therefore, the degradation trajectory is also not completely monotonous. Addressing this problem with strategy A showed some difficulties, particularly trying to forecast the dynamic or trend of the signal when the trend of the segment changes in the opposite sense to the degradation, obtaining an overestimation of the RUL. Working with this strategy showed that only the Prophet approximates the ground truth, but it is still not good enough and acceptable. From the strategy B perspective and using the RUL predictive model transferred from the IFP setting provided better results compared to the previous strategy, converging to the ground truth as it reaches the EoL with a few minor exceptions.

For the three problems addressed in this work, the degradation signals present irregularities that affect the forecast of the dynamic of the signal by a fitted model; even with Prophet, which is based on time series decomposition, it could not handle these irregularities to allow a trustworthy RUL prediction to all the degradation problems.

In most of the cases, RNN models provided an underestimated RUL, opposite to the results of the linear forecasting model such as Prophet. The time spent in the prognostic process using strategy A are shown in Table 3, where we can see that ESN is the fastest method because of its simplicity in training and forecast, followed by Prophet, and finally, LSTM and GRU were similar in the time spent.

Concerning strategy B, the results showed that this strategy obtained better estimations of RULs. It seems to be robust to irregularities present in the signal, and it is helpful for problems with similar degradations and scarce historical run-to-failure signals. With this method, it is only necessary to fit the models once and simply call the best representative model by the classifier to predict the RUL, so the time spent using the fitted model to calculate the RUL is almost negligible.

Finally, two main points must be highlighted. First, the fault detection framework defined in our previous work [15] was designed from historical fault information of a pair of IFPs out of the 132 available distributed in the 66 ALMA antennas and was validated on other IFP data achieving good detection performance. Now by updating the pre-processing module in this work, it was possible to improve the robustness by reducing the sensitivity generated by the existing noise level. This was validated in other IFPs data preserving the same performance and also found that the same effect applied to other signals similar to those of IFPs can be obtained, such as the average resolution of the camera. Second, the signals that are in the clusters do not fully represent the historical signals of the IFPs; for validation purposes, some signals that were used to verify their effectiveness in the prediction of the RUL were excluded; one of them is shown in Figure 10a, the other signals showed very similar results, and most interestingly, that using the models fitted with the IFPs data it is possible to obtain a good approximation in the RUL applied to other components that have signals with similar degradations, in this case, applied to the camera resolution signal. This indicates the power of generalization that the adjusted models have against other similar problems.

## 6. Conclusions

This work shows a fault detection framework that can be transferable or scalable to other applications with similar degradation behaviors but not necessarily with the same statistical characteristics as the particular problem for which it was developed initially. Hence, it is a helpful tool because it can be used in many applications to detect faults in the system of interest without any changes in the method.

We also tested the performance of RNN models and a time series decomposition model called Prophet to measure the precision of the RUL estimation using standard metrics proposed in [57] that allow a systematic evaluation and a level of confidence for model selection. Through this performance measurement scheme, one could eventually ask which model is the best? We argue that the best would be one that has the largest PH value and a lower $t_{\lambda }$—additionally, an underestimation of the RUL close to the ground truth. So, future works could use this as a guideline for model testing and the measurement of quality of the model used for prognostic in RUL estimation.

One of the weaknesses of this proposal in forecasting is that it depends on a catastrophic failure threshold to estimate the RUL of a component. Furthermore, it considers a deterministic threshold that could be a bit conservative if it is chosen as the worst case scenario.

## 7. Future Work

Our approach has shown to work effectively in different settings with slow degradation faults, adapting to each environment effectively. This method, together with several others that have been developed in the literature, will help organizations transform data into information. The challenge then becomes transforming this new vast information into actionable decisions. Hence, as part of our future work, we will work in:

- Improving the computation of uncertainty measurements of RUL predictions. This computation will help develop new prescriptive maintenance approaches that help in the decision-making process of maintenance procedures.
- Test this approach on other problems with similar degradation faults to continue evaluating the robustness of this run-to-failure critical segment clustering approach to predict a component’s RUL value.

## Figures and Tables

**Table 1:** Models setting used for strategy A.

**Model**						
	**ESN**		**GRU**		**LSTM**	
**Hyperparameter**	input_size:	20	input_shape:	(20, 1)	input_shape:	(20, 1)
	output_size:	1	units (GRU):	20	units (LSTM):	20
	reservoir_size:	100	activation (GRU):	reLU	activation (LSTM):	reLU
	spectralRadius:	0.75	units (Dense):	20	units (Dense):	20
	noise_scale:	0.001	activation (Dense):	reLU	activation (Dense):	reLU
	leaking_rate:	0.5	units (Dense):	1	units (Dense):	1
	sparsity:	0.3	activation (Dense):	linear	activation (Dense):	linear
	activation:	tanh	optimizer:	adam	optimizer:	adam
	feedback:	True				
	regularizationType:	Ridge				
	regularizationParam:	auto				
	**Prophet**					
	changepoint_prior_scale:			0.05		
	seasonality_prior_scale			0.01		
	daily_seasonality:			False		

**Table 2:** Models setting used for strategy B.

Model						
	**ESN**		**GRU**		**Dense**	
**Hyperparameter**	input_size:	10	input_shape:	(10, 1)	input_shape:	10
	output_size:	1	units (GRU):	15	units (Dense):	50
	reservoir_size:	250	activation (GRU):	reLU	activation (Dense):	reLU
	spectralRadius:	1.0	recurrent_dropout (GRU):	0.5	dropout:	0.5
	noise_scale:	0.001	units (GRU)	15	units (Dense):	25
	leaking_rate:	0.7	activation (GRU):	reLU	activation (Dense):	reLU
	sparsity:	0.2	recurrent_dropout (GRU):	0.5	dropout:	0.5
	activation:	tanh	units (Dense):	1	units (Dense):	1
	feedback:	False	activation (Dense):	linear	activation (Dense):	linear
	regularizationType:	Ridge	optimizer:	adam	optimizer:	adam
	regularizationParam:	0.01				

**Table 3:** Time performance measured in seconds.

Problem	Prophet	ESN	LSTM	GRU
Crack growth	252.40	109.49	2170.89	2197.84
Resolution Degradation	193.41	31.60	1995.64	1997.99
IFP Degradation	82.28	38.20	892.36	890.27

**Figure A1:**
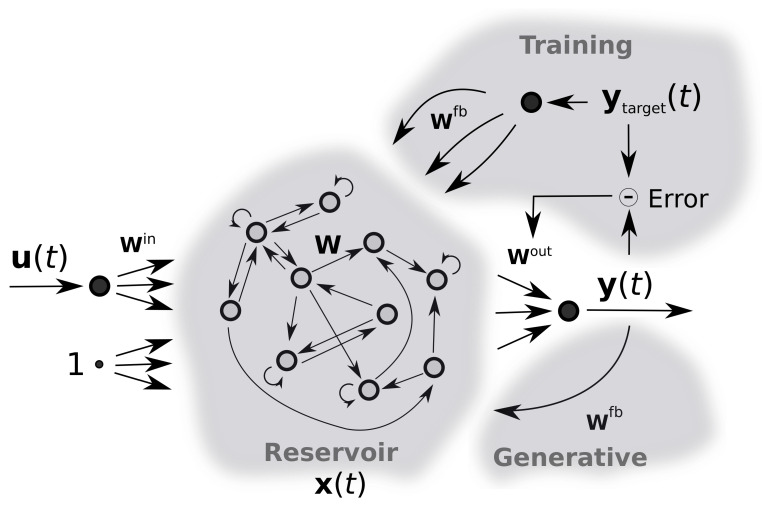
The basic echo state network architecture.

**Figure A2:**
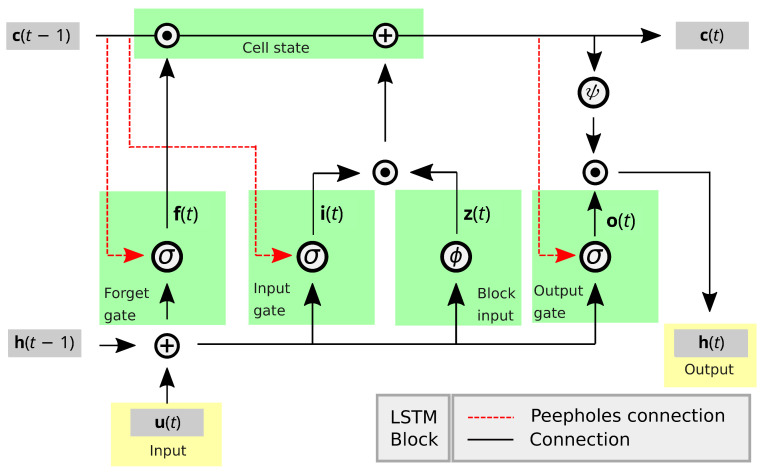
The basic LSTM architecture.

**Figure A3:**
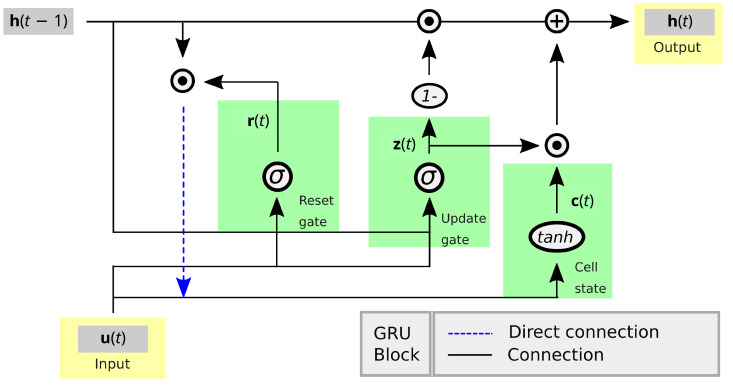
The basic GRU architecture.

## Data Availability

The data presented in this study are available on request from the corresponding author.

## Abbreviations

The following abbreviations are used in this manuscript:

RUL	**R**emaining **U**seful **L**ife
RNN	**R**ecurrent **N**eural **N**etwork
ALMA	**A**tacama **L**arge **M**illimeter **A**rray
CBM	**C**ondition-**B**ased **M**aintenance
PHM	**P**rognostic and **H**ealth **M**anagement
PH	**P**rognostic **H**orizon
EoL	**E**nd-**o**f-**L**ife
ESN	**E**cho **S**tate **N**etwork
LSTM	**L**ong-**S**hort **T**erm **M**emory
GRU	**G**ated **R**ecurrent **U**nit
ADTK	**A**nomaly **D**etection **T**ool**k**it
MVM	**M**inimum **V**ariance **M**atching
IFP	**I**ntermediate **F**recuency **P**rocessor
LSB	**L**ower **S**ide**b**and
USB	**U**pper **S**ide**b**and
SW	**Sw**itch matrix current
UT	**U**nit **T**elescope
CCD	**C**harge-**C**oupled **D**evice
EoP	**E**nd-**o**f-**P**rediction
ANN	**A**rtificial **N**eural **N**etwork
SP	**S**hortest **P**ath

## Appendix A. Evaluation Metrics

Let J be the set of all time indexes when the prediction is made, $r_{*}$ the ground truth Remaining-Useful-Life (RUL), α is the allowable error bound, $t_{P}$ time when the first prediction is made, $t_{i}$ time at the time index *i*, and EoP as End-of-Prediction of the RUL.

- **Pronostic Horizon (PH)**: it identifies whether a method predicts within specified limits around the ground truth End-of-Life (EoL) so that the predictions are considered trustworthy. If it does, how much time does it allow for any maintenance action to be taken. The longer PH better the model and more time to act based on the prediction with some desired credibility. This metric is defined as:
  (A1) $$PH=t_{EoL}-t_{i},$$
  where
  $$i=min\left\{j|\left(j\in J\right)\wedge \left[\varrho_{\alpha }^{-}\leq r\left(j\right)\leq \varrho_{\alpha }^{+}\right]\right\},$$
  $$\begin{array}{ccc} \varrho_{\alpha }^{-} & = & r_{*}-\alpha \cdot t_{EoL},\\ \varrho_{\alpha }^{+} & = & r_{*}+\alpha \cdot t_{EoL}. \end{array}$$
- **α–λ Accuracy**: this metric quantifies the prediction quality by identifying whether the prediction falls within specified limits at a particular time; this is a more stringent requirement as compared to PH since it requires predictions to stay within a cone of accuracy. Its output is binary since we need to evaluate whether the following condition is met,
  (A2) $$\left(1-\alpha \right)\cdot r_{*}\left(t\right)\leq r\left(t_{\lambda }\right)\leq \left(1+\alpha \right)\cdot r_{*}\left(t\right),$$
  where
  $$t_{\lambda }=t_{P}+\lambda \cdot \left(t_{EoL}-t_{P}\right).$$
- **Relative Accuracy**: a similar notion as α–λ accuracy where, instead of finding out whether the predictions fall within given accuracy levels at a given time $t_{\lambda }$, we also quantitatively measure the accuracy by the following
  (A3) $$RA_{\lambda }=1-\frac{r_{*}\left(t_{\lambda }\right)-r_{t_{\lambda }}}{r_{*}\left(t_{\lambda }\right)},$$
  where $t_{\lambda }$ is defined previously at α–λ accuracy. For measurement of the general behavior of the algorithm over time, Cumulative Relative Accuracy (CRA) can be used, and it is defined as
  (A4) $$CRA_{\lambda }=\frac{1}{\left|J_{\lambda }\right|}\sum_{i=1}^{J_{\lambda }}w\left(r\right)RA_{\lambda }$$
  where w(r) is a weight factor as a function of the RUL at all time indices, $J_{\lambda }$ is the set of all time indexes before $t_{\lambda }$ when a prediction is made, and |·| is the cardinality operation of a set. The meaning of these metrics is that as more information becomes available, the prognostic performance improvement will increase as it converges to the ground truth RUL.
- **Convergence**: it is a useful metric since we expect a prognostics algorithm to converge to the true value as more information accumulates over time. Besides, it shows that the distance between the origin and the centroid of the area under the curve for a metric quantifies convergence, and a faster convergence is desired to achieve high confidence in keeping the prediction horizon as large as possible. Lower distance means a faster convergence. The computation of this metric is defined as, let $\left(x_{c},y_{c}\right)$ be the center of mass of the area under the curve M(i). Then, the convergence $C_{M}$ can be represented by the Euclidean distance between the center of mass and $\left(t_{P},0\right)$, where
  $$C_{M}=\sqrt{{\left(x_{c}-t_{P}\right)}^{2}+y_{c}^{2}},$$
  $$x_{c}=\frac{1}{2}\frac{\sum_{i=P}^{EoP}\left(t_{i+1}^{2}-t_{i}^{2}\right)M\left(i\right)}{\sum_{i=P}^{EoP}\left(t_{i+1}-t_{i}\right)M\left(i\right)},$$
  $$y_{c}=\frac{1}{2}\frac{\sum_{i=P}^{EoP}\left(t_{i+1}-t_{i}\right)M^{2}\left(i\right)}{\sum_{i=P}^{EoP}\left(t_{i+1}-t_{i}\right)M\left(i\right)},$$
  M(i) is a non-negative prediction error accuracy or precision metric. In other words, this metric measures the fastness of convergence of a method.

## Appendix B. Recurrent Neural Networks

### Appendix B.1. Echo State Networks (ESNs)

The ESNs are a type of recurrent neural network developed by Herbert Jaeger [76] that has a dynamical memory to preserve in its internal state a nonlinear transformation of the input’s history. Hence, they have shown to be exceedingly good at modeling nonlinear systems. Another advantage of ESNs is that they are easy to train because they do not need to backpropagate gradients as classical ANNs do.

An ESN can be defined as follows: consider a discrete-time neural networks like in [76,77,78,79], with $N_{u}$ input units, $N_{x}$ internal units (also called reservoir units), and $N_{y}$ output units. Activations of input units at time step *t* are $u\left(t\right)\in {I\;R}^{N_{u}}$, of internal units are $x\left(t\right)\in {I\;R}^{N_{x}}$, and of output units $y\left(t\right)\in {I\;R}^{N_{y}}$. The connection weight matrix $W^{\mathrm{in}}\in {I\;R}^{N_{x}\times \left(1+N_{u}\right)}$ for the input weights, $W\in {I\;R}^{N_{x}\times N_{x}}$ for reservoir connections, $W^{\mathrm{out}}\in {I\;R}^{N_{y}\times \left(1+N_{u}+N_{x}\right)}$ for connections to the output units, and $W^{fb}\in {I\;R}^{N_{x}\times N_{y}}$ for the connections that are projected back (also called feedback) from the output to the internal units. The connections go directly from input to output units and connections between output units are allowed. Figure A1 shows the basic network architecture.

The activation of reservoir units are represented by

(A5) $$\begin{array}{cc} \tilde{x}\left(t+1\right) & \;=\;\tanh\left(W^{\mathrm{in}}\left[1;u(t+1)\right]\right.\\ & \;\;\;\;\left.+Wx\left(t\right)+W^{\mathrm{fb}}y\left(t\right)\right), \end{array}$$

and are updated according to

(A6) $$x\left(t+1\right)=\left(1-\delta \right)x\left(t\right)+\delta \tilde{x}\left(t+1\right),$$

where δ∈(0,1] is the leaky integrator rate. The output is calculated by

(A7) $$y\left(t+1\right)=W^{\mathrm{out}}\left[1;u(t+1);x(t+1)\right],$$

where [·;·] denotes the vertical vector concatenation. The coefficients in $W^{\mathrm{out}}$ are computed by using ridge regression, solving the following equation,

(A8) $$Y_{target}=W^{\mathrm{out}}X,$$

where $X\in {I\;R}^{(1+N_{u}+N_{x})\times T}$ with columns $\left[1;u(t);x(t)\right]$ for t=1,…,T; and all x(t) are produced by presenting the reservoir with u(t) and $Y_{target}\in {I\;R}^{N_{y}\times T}$.

Finally, the solution can be represented by

(A9) $$W^{\mathrm{out}}=Y_{target}X^{T}\left(XX^{T}+\tau I\right),$$

where $I\in {I\;R}^{\left(1+N_{u}+N_{x}\right)\times \left(1+N_{u}+N_{x}\right)}$ is the identity matrix and τ is a regularization factor (ridge constant). The ridge constant is estimated using grid search and time series cross-validation methods.

### Appendix B.2. Long-Short Term Memory (LSTM)

LSTM is another type of artificial recurrent neural network (RNN) architecture proposed by Hochreiter and Schmidhuber [80] that deals with the vanishing gradient problem. One LSTM unit is composed essentially of three gates: an input gate, an output gate, and a forget gate; and a memory cell that remembers values over arbitrary time intervals, and the three gates regulate the flow of information into and out of the cell. This type of RNN has been found extremely successful in many applications [81] and was regarded as one of the most popular and efficient RNN models using back-propagation as a training method. A typical LSTM [82] is illustrated in Figure A2, and can be formulated as follow.

Let $u\left(t\right)\in {I\;R}^{N_{u}}$ an input vector at time *t*, and consider *M* of LSTM units, then

- **Block input**: it consists of combining the input u(t) and the previous output of LSTM units h(t−1) for each time step *t*, and it is defined as
  (A10) $$z\left(t\right)=\phi \left(W_{z}u\left(t\right)+R_{z}h\left(t-1\right)+b_{z}\right).$$
- **Input gate**: this gate decides which values needs to be updated with new information to the cell state. It is computed as a combination of the input u(t), the previous output of LSTM units h(t−1), and the previous cell state c(t−1) for each time step *t*,
  (A11) $$\begin{array}{ccc} i(t) & = & \sigma \left(W_{i}u\left(t\right)+R_{i}h\left(t-1\right)\right.\\ & & \;\;\left.+p_{i}⊙c\left(t-1\right)+b_{i}\right). \end{array}$$
- **Forget gate**: it makes the decision of what information needs to be removed from the LSTM memory, and it is calculated similarly to the input gate.
  (A12) $$\begin{array}{ccc} f(t) & = & \sigma \left(W_{f}u\left(t\right)+R_{f}h\left(t-1\right)\right.\\ & & \;\;\left.+p_{f}⊙c\left(t-1\right)+b_{f}\right). \end{array}$$
- **Cell state**: this step provides an update for the LSTM memory in which the current value is given by the combination of block input z(t), input gate i(t), forget gate f(t) and the previous cell state c(t−1).
  (A13) c(t)=z(t)⊙i(t)+c(t−1)⊙f(t).
- **Output gate**: this gate makes the decision of what part of the LSTM memory contributes to the output and it is related to the current input vector u(t), the previous output h(t−1), and the current cell state c(t).
  (A14) $$\begin{array}{ccc} o(t) & = & \sigma \left(W_{o}u\left(t\right)+R_{o}h\left(t-1\right)\right.\\ & & \;\;\left.+p_{o}⊙c\left(t\right)+b_{o}\right). \end{array}$$
- **Block output**: finally, this step computes the output h(t), which combines the current cell state c(t) and the current output gate o(t).
  (A15) h(t)=ψ(c(t))⊙o(t)

In the above description, $W_{k}\in {I\;R}^{M\times N_{u}}$, $R_{k}\in {I\;R}^{M\times M}$, $p_{k}\in {I\;R}^{M}$, $b_{k}\in {I\;R}^{M}$, for k∈{z,i,f,o}, are input weights, recurrent weights, peephole weights, and bias weights, respectively. The operator ⊙ represent the point-wise multiplication of two vectors. $\sigma \left(x\right)=\frac{1}{1+e^{x}}$ and ϕ(x)=ψ(x)=tanh(x).

### Appendix B.3. Gated Recurrent Unit (GRU)

The GRU model was introduced by Cho et al. [83], which chose a new type of hidden unit inspired by the LSTM unit. Basically, it combines the input gate and the forget gate into a single update gate, and some operations are mixed with computing the update cell state, making this model simpler, containing fewer variables than the basic LSTM model, as shown in Figure A3. It can be formulated as follow,

Let $u\left(t\right)\in {I\;R}^{N_{u}}$ an input vector at time *t* and consider *M* of GRU units, then,

- **Update gate**: this gate determines how much previously learned information should be passed on to the future,
  (A16) $$z\left(t\right)=\sigma \left(W_{z}u\left(t\right)+R_{z}h\left(t-1\right)+b_{z}\right).$$
- **Reset gate**: this gate decides how much previously learned information to forget.
  (A17) $$r\left(t\right)=\sigma \left(W_{r}u\left(t\right)+R_{r}h\left(t-1\right)+b_{r}\right).$$
- **Cell state**: it consists of storing the relevant information from the past, using the reset gate to affect the memory content.
  (A18) $$\begin{array}{ccc} c(t) & = & \tanh\left(W_{c}u\left(t\right)\right.\\ & & \;\left.+R_{c}h\left(t-1\right)⊙r\left(t\right)+b_{c}\right). \end{array}$$
- **Block output**: finally, compute the output y(t)
  (A19) $$h\left(t\right)=c\left(t\right)⊙z\left(t\right)+h\left(t-1\right)⊙\left(1-z(t)\right)$$

In the above description, $W_{k}\in {I\;R}^{M\times N_{u}}$, $R_{k}\in {I\;R}^{M\times M}$, $b_{k}\in {I\;R}^{M}$, for k∈{z,r,c}, are update gate weights, reset gate weights, cell state weigths, and bias weights, respectively. The operator ⊙ represent the point-wise multiplication of two vectors, and $\sigma \left(x\right)=\frac{1}{1+e^{x}}$.
